# New Members for the I. H. A.

**Published:** 1888-11

**Authors:** S. A. Kimball

**Affiliations:** Secretary I. H. A.; Boston, Mass., 124 Commonwealth Avenue


### NEW MEMBERS FOR THE I. H. A.

(In reprinting the following circular from the Secretary of the I. H. A., we earnestly commend the subject to our readers. All true homœopaths should join this Association and aid in building up Homœopathy; at least five hundred of our readers should join.)

DEAR DOCTOR:—If you know of any physician who practices Homœopathy in accordance with the principles of Hahnemann, and for whom you can vouch, you are requested to ask him or her to make application for membership in the International Hahnemannian Association.

It is not desirable to increase our membership for the sake of numbers alone, *quality* not *quantity* being the object sought; but it is known that there are many good homœopathists not yet members, whom we should have with us.

Your attention is called to the extracts from the By-laws on the inclosed application blanks, which must be strictly adhered to.

The applications for membership should be sent to Dr. J. A. BIEGLER, 58 South Clinton Street, Rochester, N. Y., on or before December 1st, 1888, and theses of the applicants must also be sent to him not later than April 20th, 1889.

Very truly yours,
S. A. KIMBALL,
*Secretary I. H. A.*

BOSTON, MASS., 124 Commonwealth Avenue.

